# Editorial: Pharmacokinetic Evaluation and Modeling of Clinically Significant Drug Metabolites

**DOI:** 10.3389/fphar.2021.693922

**Published:** 2021-05-20

**Authors:** Constantin Mircioiu, Valentina Anuta, Momir Mikov, Adrian Nicolescu, Victor A. Voicu

**Affiliations:** ^1^ Faculty of Pharmacy, “Carol Davila” University of Medicine and Pharmacy, Bucharest, Romania; ^2^ Faculty of Medicine, University of Novi Sad, Novi Sad, Serbia; ^3^ Department of Medicine, Queen’s University, Kingston, ON, Canada

**Keywords:** IVIVC, metabolites, biorelevant dissolution testing, first pass metabolism, pharmacokinetics

Pharmacokinetic evaluations and modeling in order to correlate in vitro drug dissolution kinetics with their in vivo release and absorption kinetics, estimated from the deconvolution of their pharmacokinetic data, is known as IVIVC. IVIVC is a main tool in the development of new drug formulations, and is required by regulators in the case of extended-release drugs, following well-established procedures that have subsequently been tried in applications for immediate-release medicinal products (Emami, 2010; Cardot and Davit, 2012). Additionally, successful IVIVCs allow subsequent waivers of in vivo studies for bioequivalence (Karalis et al., 2010).

In most cases, applications of such correlations for immediate release lipophilic drugs encounter difficulties due to slow and incomplete dissolution from dosage forms, extensive metabolism, distribution in deep compartments, and enterohepatic circulation (Chrenova et al., 2010).

Recently, an alternative IVIVC method for such drugs has been proposed: the correlation between the dissolution kinetics of parent drugs *in vitro* and the plasma kinetics of their metabolites (Mircioiu et al., 2019), mainly due to the fact that metabolites have a simpler pharmacokinetic model (Marchidanu et al., 2013). On the other hand, the performance of IVIC is dependent on the results of in vitro dissolution methods. It is necessary to model the release kinetics and establish the most biorelevant method (Cardot and Davit, 2012; Preda et al., 2012; Paolino et al., 2019).

The use of metabolites PK in the evaluation of bioequivalence is also a challenge. There are critical situations that cannot be solved without an evaluation of the metabolites if: 1) the parent drug levels in plasma are too low to allow accurate analytical measurements, 2) the parent drug is unstable in the biological matrix, 3) the parent drug is an inactive prodrug, 4) the formation of the metabolite occurs rapidly, or 5) the metabolite significantly contributes to the overall net activity.

In the frame of this research topic, contributions have been received concerning:—Development of biorelevant in vitro dissolution tests

- Modeling of the PK of metabolites.
- Use of PK of metabolites in the evaluation of bioequivalence—time scaling for IVIVC.
- Drug-drug pharmacokinetic interactions.

Li et al. studied the influence of danhong injection, a mixture of Salvia miltiorrhiza and Carthamus tinctorius, on the PK of acetylsalicylic acid (ASA), using an analysis based on 5,183 clinical cases by determining the PK and disposition profile of salicylic acid (SA), the primary metabolite of ASA in rats. The maximum plasma concentration of SA increased significantly, by a factor of 1.37, while renal excretion of SA significantly decreased by 32.54%.

Huang et al. determined the toxicology and PK of simvastatin in cerebrospinal fluid after intradiscal injection in rabbits.

Gao et al. studied the in vitro metabolic stability and in vivo oral bioavailability of a novel compound, YL-IPA08. It was determined how metabolic disposition by microsomal P450 enzymes in the liver and intestine limited its bioavailability and further affected brain penetration to the target. An extensive first-pass effect was found in the gut (35%) and liver (17%). Therapeutic human plasma concentrations were predicted to be 27.2 ng/ml.

Li et al. studied HY-021068, which is under development as an anti-platelet agent. Plasma concentrations of HY-021068 and its effects on the platelet aggregation rate were determined. A one-compartment model with saturable Michaelis-Menten elimination was fitted to the pharmacokinetic data of HY-021068.

You et al. have shown that nicotinamide mononucleotide (NMN), a key precursory metabolite of NAD+, is able to elevate the cellular level of NAD+ and ameliorate various age-related diseases in mice and beagles.

Liu et al. studied the disposition of formononetin via the sulfonation pathway.

The expression-activity correlation was performed to identify the contributions of sulfotransferase to formononetin metabolism. Human embryonic kidney cells catalyzed formononetin formation of a monosulfate metabolite. Sulfate formation of formononetin in the cell lysate followed Michaelis-Menten kinetics.

Rizea-Savu et al. developed an analytical method for the determination of alverine, in combination with the metabolites 4-hydroxy alverine, N-desethyl alverine, and 4-hydroxy alverine glucuronide, in human plasma. The PK were determined in an open label, non-comparative study of Spasmonal® Forte. It was found that the parent compound, alverine, is subject to high PK variability, the metabolic process most susceptible to outlying performance is hydroxylation to the active metabolite 4-hydroxy alverine. Another observation was that alverine accounts for only 3% of alverine-related moieties in circulation, whereas total 4-hydroxy alverine accounts for 94%.

Shleghm et al. developed a method for estimating the in vivo release of amiodarone from the PK of its active metabolite, desethylamiodarone, and correlation with its in vitro release. The correlation of the in vitro dissolution and estimated in vivo dissolution of amiodarone was based on a model proposed by the authors that considers that amiodarone has a slow dissolution, rapid absorption, and rapid metabolism and, before returning to the blood from other compartments, its PK is determined mainly by the kinetics of release in the intestine from the pharmaceutical formulation. Under these conditions, the rate of apparition of desethylamiodarone in the blood was found to be a metric of the release of amiodarone in the intestinal fluid.

Yang et al. developed a novel intravenous formulation for general anesthesia by encapsulating isoflurane molecules into an emulsion. A clinical study demonstraed that the bioequivalence of emulsified and non-emulsified isofluarane; the safety and anesthesia effectiveness were also similar.

Ghiciuc et al. demonstrated that the low solubility and high permeability of amiodarone is the limiting step for its bioavailability, therefore, new formulations are needed that improve the solubility of amiodarone, either to increase its oral bioavailability or to reduce its toxic effects. A study on the acute toxicity of amiodarone using new complexes with cyclodextrin demonstrated that including amiodarone in cyclodextrin does not lead to increased toxicity.
